# A Case Report of Rare Bilateral Quadriceps Tendon Rupture Diagnosed by Point of Care Ultrasound (POCUS)

**DOI:** 10.5070/M5.52334

**Published:** 2026-07-31

**Authors:** Joel Hines, Christopher Blanton, Jodi Jones, Prayag Mehta

**Affiliations:** * UT Southwestern Medical Center, Department of Emergency Medicine, Dallas, TX

## Abstract

A 49-year-old male presented to the emergency department (ED) with bilateral knee pain that began after a mechanical fall down several steps. He states that he fell in such a way that both knees were flexed while he was in the air, which he described as a “flying prayer” position, and he landed with all his weight onto his flexed knees. He did not immediately seek medical care but instead wore knee braces and used crutches to ambulate. This resulted in multiple other falls due to persistent pain and lack of mobility. He was seen at an outside facility prior to presenting to our ED and was reportedly diagnosed with severe arthritis based on x-ray imaging and told he may have a minor quadriceps tendon injury. No other imaging was performed at that time. Due to continued pain, he presented to our ED where we utilized point-of-care ultrasound (POCUS) which revealed the presence of bilateral full-thickness quadricep muscle and tendon tears. He was treated with surgical repair of his quadriceps tendons and had an uncomplicated postoperative course. This case report highlights the importance of ultrasound in diagnosing musculoskeletal injuries and improving patient outcomes. We also summarize POCUS findings that emergency physicians can use to diagnose quadriceps muscle and tendon tears.

**Topics:**

Point-of-care ultrasound (POCUS), musculoskeletal ultrasound, tendon rupture, fall. Hines J, et al. A Case Report of Rare Bilateral Quadriceps Tendon Rupture Diagnosed by Point of Care

**Figure:**
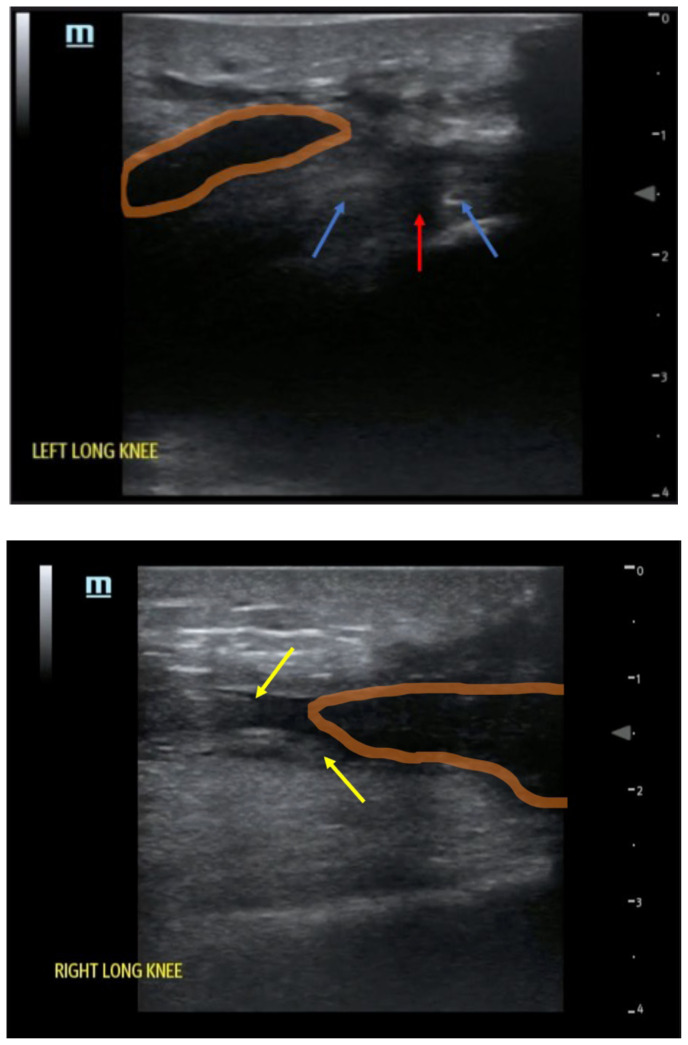
Video Link: https://youtu.be/GQlT3kkWwPM

## Brief introduction

Quadriceps tendon rupture (QTR) is a rare but serious injury that should be rapidly identified in the ED for the best functional outcomes.^1^ The incidence of QTR is relatively low, as stated in the literature, at 1.37/100,000 people/year.^2^,^3^ The incidence of unilateral rupture is significantly more common than bilateral ruptures.^4^,^5^ Often, QTR results from an eccentric contraction of the quadriceps while the knee is in partial flexion.^3^ Partial ruptures can retain some function, which can obscure the clinical diagnosis of rupture. Given its potential for misdiagnosis, timely recognition through clinical examination and imaging is essential.^1^ While magnetic resonance imaging (MRI) remains the gold standard for diagnosis, POCUS is an accessible, fast, highly sensitive, and cost-effective alternative increasingly being utilized in the ED.^4^ This case study explores a patient presentation of bilateral traumatic QTR diagnosed in the emergency department using POCUS and examines the advantages, limitations, and clinical implications of POCUS for diagnosing QTR.

## Presenting concerns and clinical findings

The patient is a 49-year-old male with no prior past medical history. He presented to the ED for bilateral knee pain that was persistent and progressive after a recent injury. He reported falling down three-four stairs in a position he called the “flying prayer,” in which he had bilateral knee hyperflexion and both of his hands in the air when he landed on the ground. His weight was directly distributed onto his knees in that position with the fall. He suffered from limited lower extremity mobility secondary to significant pain and swelling, leading to a series of falls that required him to wear knee braces and to ambulate with crutches. His most recent fall was a few days prior to arrival at our ED. He was seen at an outside hospital and, after review of their ED notes, was told he had arthritic changes on his knee x-rays and concern for minor bilateral quadriceps tendon injury, managed conservatively. However, no other imaging was performed at that time.

The physical exam in our ED was remarkable for bilateral knee ecchymoses and severe pain with knee extension bilaterally that was worse on the right. He also had significant suprapatellar tenderness to palpation with palpable suprapatellar defects bilaterally. An extension lag, the inability to fully extend, was noted in the left lower extremity. Sensory function remained grossly intact. Compartments were soft and compressible. Distal pulses were easily palpable. Knee x-rays performed in our ED revealed small suprapatellar joint effusions bilaterally but no fractures or dislocations.

## Significant findings

Musculoskeletal assessment with POCUS revealed disruption of the left quadriceps tendon (blue arrows) indicated by a large hypoechoic gap (red arrow) with significant surrounding fluid throughout the quadriceps as well as a sizable left knee effusion (orange highlighting). The right lower extremity had a disrupted and retracted quadriceps tendon (yellow arrow) with associated fluid near the injury site and a small right knee joint effusion (orange highlighting). Therefore, he was diagnosed with bilateral quadriceps tendon ruptures.

Musculoskeletal and soft tissue ultrasound is best performed with the high-frequency linear transducer (10–15 MHz) for optimizing superficial soft tissue structures. Place your patient in a supine position with the knee slightly flexed to relax the quadriceps muscles. Place a rolled towel or pillow under the knee to help maintain this position comfortably. Begin with the probe in the longitudinal orientation over the anterior knee, just superior to the patella. Identify the patella inferiorly and the quadriceps tendon superiorly. In healthy patients, the tendon appears as a homogeneous, fibrillar structure attaching to the superior pole of the patella. Asking the patient to gently contract the quadriceps, if tolerated, may help demonstrate discontinuity or abnormal tendon movement. An intact quadriceps tendon appears continuous, with parallel echogenic fibers spanning from the muscle belly to the patella. There should be no fluid collection or hypoechoic gaps.

Findings suggestive of QTR include hypoechoic or anechoic gaps within the tendon fibers (Figure 1). Other findings include heterogeneous or disorganized tendon architecture, hematoma or fluid collection in the suprapatellar region, or retraction of the tendon ends (Figure 2). Some pitfalls to avoid in assessing for QTR with POCUS include mistaking anisotropy for pathology; therefore, keep the probe angle perpendicular to the tendon fibers. Another common error is incomplete scanning. Evaluate in both long and short axes and compare with the contralateral knee if uncertain.

## Patient course

This patient could have been managed as an outpatient but was admitted to the hospital after he failed a physical therapy evaluation in the ED. He lived on the third story of an apartment complex without elevators and was unable to climb stairs during his evaluation. The next day, he had an MRI which confirmed the diagnosis of bilateral QTR. More specifically, MRI revealed a torn left distal rectus femoris tendon at the patellar attachment, a complete or near complete proximal tear of the left vastus medialis attachment to the femur, a complex tear of the right quadriceps musculature, and a retracted distal rectus femoris musculotendinous junction.

He underwent prompt bilateral surgical repair of the quadriceps tendons, which revealed shearing injuries at the superior pole of both patellae. Overall, his postoperative course was uncomplicated. He remained afebrile, tolerated a regular diet, received physical therapy and occupational therapy, achieved pain control with oral medications, and ambulated with a walker. Post-operative recommendations included bilateral lower extremities being locked in extension for 12 weeks, increased range of motion to 0–20 degrees after two weeks, and subsequent progression of 10-degree increments weekly. He was discharged to our physical medicine and rehabilitation (PM&R) unit. After significant improvement in his strength, function, and mobility, he was discharged home after 15 days in the PM&R unit. At the time of discharge, our patient was ambulating with bilateral knee braces in extension and a walker. He was now able to climb three stories of stairs independently. He did complain of some bilateral knee discomfort, but pain was well controlled with conservative analgesia, including acetaminophen and methocarbamol, as well as oxycodone for moderate-to-severe pain that he rarely used.

## Discussion

Quadriceps tendon rupture is an uncommon injury in ED patients, with bilateral QTR being exceedingly rare. There is literature that correlates a predisposition for QTR with pre-existing degenerative changes secondary to systemic conditions like diabetes, gout, or corticosteroid and fluoroquinolone use, which can weaken the tendon and accelerate degeneration.^2^ Of note, this patient had an elevated glucose of 316 mg/dL upon initial laboratory workup and a hemoglobin A1C of 7.5%, leading to a new diagnosis of type 2 diabetes mellitus. Prompt recognition, diagnosis, and management of QTR is crucial for ensuring recovery of maximal function and preventing complications such as quadriceps atrophy and tendon retraction that can develop with prolonged time to definitive treatment. Clinical examination remains a cornerstone of diagnosis for QTR. Studies indicate that with examination alone, up to 50–67% of cases are misdiagnosed at initial presentation.^2^,^3^ Typical clinical findings of QTR include loss of active leg extension, knee pain, swelling, and a palpable suprapatellar gap. The sensitivity of physical examination alone is limited due to factors such as swelling and retention of partial function, particularly in cases of partial rupture where some degree of function may be retained.^2^,^3^

Imaging modalities such as radiographs, MRI, and ultrasound play an essential role in clinching the diagnosis of QTR. Each of these radiographic studies has their own distinct advantages and disadvantages. X-rays are fast and easily accessible in most emergency departments, but the amount of information they can provide is limited at best without the ability to provide detail regarding soft tissue injuries. The gold standard for diagnostic imaging of QTR is MRI. It is highly accurate and can provide great detail to assess the extent of the injury and damage to the surrounding structures. While MRI remains one of the best options for its high sensitivity and specificity for QTR diagnosis, it may not be the best first option for providers in the emergency department.^1^,^3^,^6^ Many emergency departments have limited availability of MRI’s. Even amongst facilities with MRI availability, they typically will have a longer wait due to scheduling and longer exam durations associated with MRI. With the higher cost to the patient, the possibility of limited access, and longer acquisition time, MRI is frequently a less practical option for emergency department care. Conversely, POCUS is a tool that most ED providers are familiar with and use on a daily basis. Many studies have shown that ultrasound is an effective diagnostic tool for the diagnosis of soft tissue injury, including QTR.^3^,^6^,^7^ Assessment with POCUS can allow for rapid diagnosis for a fraction of the time and cost of obtaining an MRI. One of the main advantages of POCUS is its ability to perform dynamic assessments. During a dynamic assessment, the operator can visualize and assess the integrity of the QTR in real time during movement of the knee to elucidate the presence of partial tears further. Finally, with POCUS the operator can directly assess the patient’s point of maximal pain and quickly compare the unaffected extremity for a baseline comparison.^2^,^8^

Despite all the advantages of POCUS for diagnosing QTR at the bedside, ultrasound has its limitations. A retrospective study comparing the use of MRI versus ultrasound in 66 patients for diagnosis of QTR in patients who were ultimately brought to the operating room for repair revealed that the sensitivity of MRI and POCUS were both 1.0. In this study, MRI retained a specificity of 1.0 and a positive predictive value of 1.0, while POCUS had a specificity of 0.62 and a positive predictive value of 0.88.^3^ In this study, the ultrasounds were performed by musculoskeletal trained radiologists. To date, there are no studies evaluating sensitivity in ultrasounds performed by emergency physicians. Given that ultrasound is an operator-dependent diagnostic study, there is potential for false positives. Phenomena such as anisotropy can lead more inexperienced operators to interpret that a tear is present when the tendon is actually intact.^7^ Given the limitations of POCUS described above, many studies have recommended that positive POCUS exams be followed up with MRI to confirm findings and prevent the possibility of surgical intervention on intact tendons with false positive exam findings.^1^,^3^ Since the sensitivity of POCUS is equal to MRI, with the added benefit of high availability, low patient cost, and short time to acquisition, the use of POCUS as a frontline imaging modality in the emergency department to screen for QTR in patients with clinical suspicion can help reduce time to diagnosis and definitive management.

Given the potential consequences of poor functional outcomes that accompany delayed recognition and treatment of QTR, especially in the case above with bilateral QTR, utilizing bedside POCUS for rapid evaluation of the presence of QTR is an accurate and efficient use of resources. With the integration of POCUS as a screening tool for QTR injury, providers can expedite diagnosis and consultation of the appropriate teams for definitive management. After a thorough literature review, our recommendation is to adopt a stepwise approach including a thorough clinical exam followed by a POCUS screening for tendon rupture. Any positive findings may need to be followed by an MRI for confirmation of pathology before the decision for surgery is made. This case study highlights how POCUS can be safely, accurately, and efficiently incorporated into the emergency department workflow as a widely available, non-invasive diagnostic tool for rapid diagnosis of QTR, lowering costs to patients, expediting treatment decisions, and enhancing patient care.

## Supplementary Information










